# HIV among people who inject drugs in Hungary

**DOI:** 10.1186/s40249-017-0360-9

**Published:** 2017-10-11

**Authors:** András Ortutay, V. Anna Gyarmathy, Zsuzsa Marjanek, Károly Nagy, József Rácz, István Barcs

**Affiliations:** 1 Anaesthesiology and Intensive Care Unit, Jávorszky Ödön Hospital, Vác, Hungary; 2 Johns Hopkins Bloomberg School of Public Health, Baltimore, MD USA; 3 Faculty of General Medicine, Institute of Medical Microbiology, Semmelweis University, Budapest, Hungary; 4 Institute of Psychology, Eötvös Loránd University, Budapest, Hungary; 5 Faculty of Health Sciences, Department of Addictology, Semmelweis University, Budapest, Hungary; 6 Faculty of Health Sciences, Department of Epidemiology, Semmelweis University, Budapest, Hungary

**Keywords:** People who inject drugs, HIV/AIDS, Surveillance

## Abstract

**Background:**

Before 2014 (the year of closure of the two largest needle exchange programs in Hungary, which halved the number of available syringes in the country despite increased injecting risk practices) no HIV was reportedly acquired in Hungary among people who inject drugs (PWIDs) who were not also men who had sex with other men (MSM). In 2014, one and in 2015 two non-MSM PWIDs were newly diagnosed with HIV who supposedly became infected in Hungary, and both incident HIV cases in 2015 were diagnosed in the AIDS stage. In addition, two new (albeit supposedly imported) non-MSM PWID cases were also registered in the first three quarters of 2016, one of which subsequently was diagnosed with and then died of AIDS. At the same time, the prevalence of HCV doubled among PWIDs (from 24% to 49% in Hungary and from 34% to 61% in Budapest).

**Case presentation:**

The case that we discuss in this paper is a male PWID, who was diagnosed with HIV and AIDS in May of 2015 and then died of AIDS the next month. His HIV infection status was detected with delay, and then appeared in the official statistics as an incident PWID HIV case and an incident PWID AIDS case, but not as an incident PWID AIDS death. No contact tracing followed, even though it would have been relatively easy considering the circumstances. To our knowledge, no HIV post-exposure protocol exists in hospitals, in case of HIV exposure due to an eventual needle-stick injury.

**Conclusions:**

Our paper draws attention to recently published HIV and AIDS surveillance data, and shows the failure of the system. While sounding the alarm based on three newly detected PWID HIV cases in the past 2 years may be premature, there are definitely serious problems in the HIV detection and tracing system among PWIDs in Hungary.

**Electronic supplementary material:**

The online version of this article (10.1186/s40249-017-0360-9) contains supplementary material, which is available to authorized users.

## Multilingual abstracts

Please see Additional file 1 for translations of the abstract into the five official working languages of the United Nations.

## Background

**Fig. 1:**
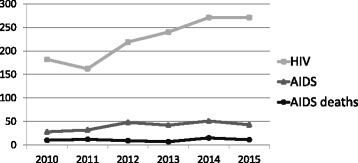
HIV and AIDS incidence in Hungary in the general population, as of December 31, 2015. Data based on surveillance conducted by the National Center for Epidemiology between 2010 and 2015. Note: numbers refer to the total number of cases within the country

Recently released surveillance data in Hungary (a country with about 10 million inhabitants) show low incidence of HIV and AIDS (respectively 271 and 43 new cases in 2015 and a cumulative total of 3291 and 897 cases by the third quarter of 2016) (Fig. 1) [1]. Although only 28 people who inject drugs (PWIDs) were registered with HIV infection, the situation relating to PWIDs may be a cause for concern. Before 2014, there were two PWID HIV cases who were also men who had sex with men (MSM) who self-reported possibly having become infected in Hungary sexually, but all 21 other infected PWIDs who were not MSM confirmedly contracted HIV abroad [2]. However, in 2014 one and in 2015 two new cases of HIV were reported among PWIDs who – according to surveillance documentation – were infected in Hungary [1]. In addition, the two new PWID HIV cases in 2015 were identified in the AIDS stage. No incident PWID AIDS deaths appeared in the official statistics in 2015. In addition, two new (albeit supposedly imported) non-MSM PWID cases were also registered in the first three quarters of 2016, one of which subsequently was diagnosed with and then died of AIDS [1]. By presenting one of the cases and the surrounding circumstances, the authors will illustrate the need to reassess the current situation.

## Case presentation

A man in his mid-twenties who lived in a small village not far from Budapest presented in May of 2015 to the emergency room of a hospital near him with severe fatigue, dizziness and drowsiness that had been ongoing for week. Based on the incorrect conduction of the heart he was transferred to the Cardiology Unit, where his rhythm normalized after the installation of a temporary pacemaker. The cardiological assessment was negative. Two days later a cranial magnetic resonance imaging (MRI) scan was performed, which revealed multifocal nodules possibly originating from inflammation. Subsequently he was transferred to the Intensive Therapy Unit for further workup and care. His mental status and neurological exam at that time showed altered consciousness of unclear etiology. He had a temperature of 38.7 °C (101.7 °F). Lumbar puncture revealed high amounts of total protein in the cerebrospinal fluid. Microorganisms were not detected from the patient’s blood cultures. Results of autoimmune tests were negative. Due to the patient’s partial respiratory failure, purulent discharge in the airways, and constant fever, a thoracic computed tomography (CT) scan was performed, which revealed diffuse nodular infiltrative shadows in each side of the lungs. The patient’s health deteriorated rapidly, so mechanical ventilation and antihypotensive treatment were initiated. Due to rising inflammatory parameters and a deterioration of consciousness, infective endocarditis or meningitis was suspected; therefore, antimicrobial treatment was added. The imaging and the laboratory tests ruled out the suspected diseases.

As it eventually turned out, his medical history included drug use (“herbal weed” and “injecting crystal”). Therefore, on the third day of his stay, blood samples to test for HIV were sent to a designated laboratory. The results of the HIV test, however, had not arrived even by the 7th day of the patient’s stay, so that day repeated blood samples to test for HIV were sent to the Immunology Laboratory at Szent László Hospital (this hospital is the only one in Hungary where people with HIV/AIDS are treated). On the 8th day a telephone call informed the patient’s physicians (coauthors on this paper) that his HIV test was positive, and the patient was immediately transferred to the Central Intensive Therapy Unit at Szent László Hospital. There advanced AIDS stage was diagnosed, along with a high viral load in the cerebrospinal fluid, and low CD4 percentage. Test results for opportunistic infections (*Toxoplasma, Cryptococcus*, *Mycobacterium*, EBV, and CMV IgM) were negative. A four-drug highly active antiretroviral therapy (HAART) regimen was initiated. Despite a month of treatment, the patient stayed in a coma, his MRI brain scan progressed, and he subsequently deceased.

## Discussion and conclusions

The prevention of the spread of HIV in Hungary is governed by regulations of the Hungarian Ministry of Health (regulation 18/2002. (XII. 28.) and its update of 46/2009. (XII.22.)). This regulation consists of three sections – compulsory HIV testing, voluntary HIV testing, and the process of HIV testing – and an appendix. The section on compulsory testing describes occupational groups that are regularly exposed to blood and either semen or vaginal fluids, and resolutions to situations when they refuse being tested; donations of organs, blood and maternal milk; registration and care of people with confirmed HIV infection, keeping in mind data protection and counselling about infection transmission; evaluation of infection risk following epidermal injuries and exposures of health care personnel. The section on voluntary testing outlines sexual partners of HIV infected people, children of HIV infected mothers, PWIDs, and certain migrants from third countries as groups where voluntary testing is recommended. The third section on the process of HIV testing specifies certain laboratories that are obliged to perform HIV testing, and how laboratories can be qualified to test for HIV; confidential management of personal data of those tested; confirmation of HIV testing; and how to care for HIV infected that were tested anonymously. The appendix lists up laboratories where HIV testing is performed for free, and health care providers that are authorized to care for HIV infected.

This medical case illustrates why there is a problem with the detection and management of HIV among PWIDs, including the difficulty of case identification, delay of diagnosis, and lack of post-exposure prophylaxis protocol. It is notable that this hospital did not have any rapid HIV test kits: while we are not aware of any statistics on what proportion of hospitals has rapid HIV kits, given the rarity of HIV in Hungary, hospitals – including this hospital – might not be prepared for such scenarios, since many of them have never encountered it. As a matter of fact, none of the physicians that examined or treated the patient thought about HIV infection at the beginning, despite the patient’s medical history (he self-reported injecting drugs), especially because none of the typical visible AIDS symptoms (or any other visible symptoms for that matter) were present. Noteworthy problems in the system were revealed when HIV was finally suspected. Then, according to the regulation described above, blood samples were sent to a designated laboratory. Not only did this process delay proper diagnosis, but a combination of timing and common practice was also unfortunate: first, the three-day national holiday weekend following the patient’s hospital admission added further delays to the processing of the samples, and second, the laboratory – like all other laboratories in general – processed all samples in an order of arrival without differentiating between routine tests and tests needed for hospital emergencies – like this case. This was the first failure of the system.

Second, we are unaware of any national HIV post-exposure prophylaxis protocol – either because it does not exist, or because health care professionals (including the authors of this paper) are unaware of it even though it exists. While other countries have protocols, such as the compulsory testing of patients whose blood, tissue, or other body fluids that are potentially infectious got into contract with the blood or mucous membrane or non-intact skin of a health care professional and, in case of a positive HIV (or HCV or HBV) test, the immediate subsequent initiation of post-exposure prophylaxis to prevent the transmission of blood-borne viruses to the health care professional [3]. Given that in an intensive care setting, where there is often a rush to save lives, safety protocols are not always completely adhered to, situations may occur where there is an exposure to blood-borne infections. If there is no information about the serostatus of a patient in such a situation, or if testing is impossible or delayed, then post-exposure prophylaxis may not be implemented.

The third failure of the system was that after HIV was verified at the laboratory at Szent László Hospital, after the patient was transferred there and was diagnosed with AIDS, and after he died of AIDS, data showed up in the national HIV/AIDS registry only partially: while he was registered as incident PWID HIV case and an incident PWID AIDS case, he was not entered into the system as an incident PWID AIDS death. Given that HIV has been very rare in Hungary, especially among PWIDs, we suspect that his death was omitted from among the incident AIDS deaths most probably because his death certificate indicated an opportunistic illness as the cause of death instead of the underlying HIV/AIDS.

The fourth failure of the system is the subsequent lack of contact tracing. To our knowledge, no epidemiological investigation followed this patient’s identification as an HIV infected PWID, even though such contact tracing should be common practice, especially where HIV is either rare or high priority (or both) [4]. As we describe in the next paragraph, the context for an HIV epidemic might be increasingly favorable in Hungary. Therefore, contact tracing of an incident, domestically acquired HIV infection should have been conducted as a priority measure.

Even if the system had performed perfectly, however, there are some additional issues, namely with data protection, which are most probably universal. For example, if the patient had been tested for HIV before and known that he was infected, it would have been at his own discretion whether he discloses that to hospital personnel. If personnel are not prepared for HIV infected patient, that could be a problem. Since this patient lived in a small village where there are neither needle exchange programs nor any other helping programs for high-risk populations, he had a very low chance of being identified as HIV infected. Even if he had been, it would have been confidential information between him and his physician. This is a useful measure for his protection, but it could backfire in absence of general precautions in healthcare.

New psychoactive substances (NPS) have since about 2010 dominated the injecting drug scene in Hungary as heroin and amphetamines have become increasingly rarely available [5]. The injecting of NPS is associated with a high number of daily injecting episodes and a high prevalence of syringe sharing and reuse [2]. The Drug Prevention Foundation (DPA) opened the first needle exchange program (NEP) in Hungary in 1994 using a legal loophole, and by the end of 2014 there were altogether 31 NEPs operating in 21 cities in the country [6]. The largest in terms of number of clients, contact, and syringes distributed were Blue Point and the second largest was DPA. Despite the increased need for sterile syringes, however, these two largest NEPs were forced to close down in 2014 as a result of, among others, a politically motivated decrease of government funding [6]. The number of available syringes accordingly dropped to less than half of what it was earlier and the number of distributed syringes per PWID per year is now lower than 40% of the minimal value recommended by the WHO [6]. Therefore, not only was there limited access to NEPs in Hungary before to begin with, after the closing of the two largest programs the situation became even worse. Between 2011 and 2014 the prevalence of HCV among PWIDs rose from 24% to 49% in Hungary and from 34% to 61% in the capital city of Budapest: a situation that might be considered as a possible consequence of the closure of the NEPs [7]. Mathematical models for HIV and HCV transmission among injecting drug users delineate that HIV and HCV co-occur in a way that when the prevalence of HCV is under 35% HIV prevalence is low, but above this level the probability of an HIV epidemic increases [8]. This is why the one domestically acquired PWID HIV case in 2014 and the two cases in 2015 against a prior low number of imported cases are noteworthy, and raise the question whether these three HIV infections are isolated exceptions or whether there is a peat-fire–like hidden HIV epidemic in the making among PWIDs in Hungary. Moreover, the occurrence of a domestically acquired triple incident HIV/AIDS/AIDS death case also suggests that the case that is presented in this report may be the tip of an iceberg.

Regular screening of PWIDs for HIV and HCV has occurred through the needle exchange programs around Hungary, as a collaboration between the National Center for Epidemiology, the National Focal Point and the NEPs across the country [9]. The closure of the two largest NEPs lead to two main consequences. First, not only has the population that was served by these NEPs become largely un- or underserved in terms of injecting equipment, but they most probably will fail to participate in the annual screening campaigns for HIV and HCV. Second, since these NEPs served low socio-economic areas with very high-risk injecting profiles and high prevalence of HCV [6], the annual screening campaign will most likely fail to reach high prevalence populations in the capital, and this selection bias will most likely result in lower measured prevalence of HCV (and over time HIV) in Budapest. Therefore, if populations with high injecting risk are excluded from regular screening campaigns, an eventual HIV outbreak among PWIDs may go largely undetected.

The patient indicated injecting “crystal”, which is a common general street name for injectable NPS [5]. As such, a contact tracing exercise establishing potential routes of infection transmission should have been not only necessary because the patient was injecting drugs against the above described behavioural epidemiological and risk environment background of drug injecting in Hungary, but also relatively easy because the patient lived in a small village not far from Budapest, the capital of Hungary, where social contacts are highly visible. To our knowledge, no outbreak investigation or contact tracing followed the discovery of the second case either, even though that PWID HIV case was also detected in the AIDS stage. It seems that Hungarian public health authorities are largely unprepared or maybe even unwilling – as suggested by the government’s moralistic approach towards drug use – to deal with the reality of drug injecting related infectious diseases and related epidemics [2].

Hungary may not be the only country or area where HIV is currently rare but behavioural epidemiological and/or risk environmental background factors are such that more HIV cases might be expected at some future time. NPS – especially injectable ones – have become increasingly dominant in countries other than Hungary as well, such as Romania, Spain, the UK, Poland, Sweden and Finland [10]. While sounding the alarm based on three newly detected PWID HIV cases in the past 2 years may be premature, there are some discernible shortfalls in the HIV detection and tracing system among PWIDs in Hungary. Therefore, we have the following recommendations. First, it would be useful for the regulations to include a suggestion for hospitals to have a designated department or section where rapid HIV testing is available. Since rapid HIV test kits are neither prohibitively expensive nor bulky, they should be readily available in hospitals and clinics, and doctors should be aware of using them for patients who self-report behaviours that may put them at risk for HIV infection (especially injecting drugs or being an MSM). Second, while the regulation contains a paragraph on post exposure prophylaxis for health care professionals, having rapid HIV kits available would facilitate compliance. Third, when a person dies as a result of complications of HIV infection, HIV should be noted as the primary cause of death. Fourth, while testing of sexual and injecting contacts of HIV infected people may be voluntary, it should be compulsory for certain designated health care professionals to perform contact tracing – an aspect that is currently missing from the regulation. Therefore, contact tracing should be a priority, especially because it is a relatively manageable task due to the low HIV prevalence in the country. In general, public health authorities should do the utmost to monitor, detect and deal with infectious diseases in countries where there is evidence of substantial increases in drug injecting and syringe sharing to avoid HIV outbreaks similar to those in Greece and Romania in 2011 and 2012 [2]. In addition, protocols about post-exposure prophylaxis should not only exist, but health care personnel should be aware of them and be able to implement them. On a population level, harm reduction should be readily available for those people who engage in high-risk practices. All this, however, requires sufficient and regularly ongoing financial and moral support from the respective government and/or sponsoring entities.

## Additional file

Additional file 1: Multilingual abstracts in the five official working languages of the United Nations. (PDF 668 kb)

## Abbreviations

AIDS: Acquired Immunodeficiency Syndrome
CMV: Cytomegalovirus
CT scan: Computed Tomography scan
DPA: Drug Prevention Foundation
EBV: Epstein-Barr Virus
HAART: Highly Active Antiretroviral Therapy
HBV: Hepatitis B Virus
HCV: Hepatitis C Virus
HIV: Human Immunodeficiency Virus
IgM: Immunoglobulin M
MRI: Magnetic Resonance Imaging
MSM: Men who had sex with other men
NEP: Needle Exchange Program
NPS: New Psychoactive Substances
PWIDs: People who inject drugs
WHO: World Health Organization
