# Supplementary material for: HIV among people who inject drugs in Hungary
Source: Infect Dis Poverty. 2017 Oct 11;6:145. doi: 10.1186/s40249-017-0360-9 (PMC5635508; doi:10.1186/s40249-017-0360-9)
Supplement: Additional file 1: — Multilingual abstracts in the five official working languages of the United Nations. (PDF 668 kb) [file 40249_2017_360_MOESM1_ESM.pdf]

Translation of the abstract into the five official working languages of the United Nations

الإصابات بفيروس الأيدز بين متعاطي المخدّرات بالحقن في المجر

أندراس أورتوتاي، ف. أنا جيارماثي، سوزا مارجانيك، كارولي ناجي، جوزيف راش واستفان باركس

خلفية: قبل عام 2014 (وهو العام الذي أغلق فيه أكبر برنامجين لتبادل الحقن في المجر، مما أدى إلى خفض عدد الحقن المتاحة في البلد إلى النصف، على الرغم من زيادة ممارسات الخطرة عن طريق الحقن)، لم تحدث إصابات بفيروس الإيدزفي المجر بين الأشخاص الذين يتعاطون المخد رات بالحقن والذين لا يمارسون أيضا الجنس مع رجال آخرين. وفي عام 2014، تم تشخيص إصابة شخص وفي عام 2015 شخصين من الأشخاص إصابة حديثة من الذين يتعاطون المخد رات بفيروس العوز المناعي البشري والذين لا يمارسون الجنس مع رجال آخرين في المجر، وتم تشخيص حالتي فيروس العوز المناعي البشري في عام 2015 بأنهما في مرحلة الإصابة بالإيدز. وبالإضافة إلى ذلك، سجلت حالتان جديدتان (غير مفترض أنهما أتيتا من خارج البلد) من الذين يتعاطون المخدرات بالحقن ولا يمارسون الجنس مع رجال آخرين في الأرباع الثلاثة الأولى من عام 2016، تم تشخيص إصابتهما بعد ذلك بمرض الإيدز ومن ثم توفيا. وفي الوقت نفسه، تضاعف انتشار فيروس التهاب الكبد الوبائي بين الأشخاص الذين يتعاطون المخد رات بالحقن) من 24٪ إلى 49٪ في المجر ومن 34٪ إلى 61٪ في بودابست).

عرض حالة: والحالة التي نناقشها في هذه الورقة هي لذكر يتعاطى المخدرات بالحقن وتم تشخيص إصابته بفيروس نقص المناعة البشرية والإيدز في مايو من عام 2015، ثم توفي بسبب الإيدز في الشهر التالي. وقد تم الكشف عن حالة إصابته بفيروس نقص المناعة البشرية متأخرا، ثم ظهرت في الإحصاءات الرسمية كحالة مصابة بفيروس نقص المناعة البشرية من متعاطي المخدرات بالحقن، ولكن ليس كحالة وفاة نتيجة الإيدز. لم يتم تتبع ملابسات الحالة، على الرغم من أن هذا كان سهلا نسبيا بالنظر للظروف. على حد علمنا، لا يوجد بروتوكول بعد التعرض لفيروس نقص المناعة البشرية موجود في المستشفيات، في حالة التعرض لفيروس نقص المناعة البشرية بسبب التعرض لوخز إبرة ملوثة بالفيروس عرضا.

الاستنتاجات: توجه دراستنا الانتباه إلى بيانات مراقبة فيروس نقص المناعة البشرية والإيدز التي نشرت مؤخرا، وتبين فشل النظام. وبينما يبدو أن التنبيه المبني على ثلاث حالات اكتشف إصابتها بفيروس نقص المناعة البشرية المكتشف حديثا عن من الذين لا يمارسون الجنش مع رجال آخرين في السنتين الماضيتين قد يكون سابقا لأوانه، فإن هناك مشاكل خطيرة في نظام الكشف عن فيروس نقص المناعة البشرية وتعقبه بين الأشخاص الذين يستخدمون المخد رات بالحقن في المجر.

Translated from English version into Arabic by Mahmoud Sami, through

TRANSLATORS
WITHOUT BORDERS

匈牙利注射毒品人群的艾滋病问题

András Ortutay, V. Anna Gyarmathy, Zsuzsa Marjanek, Károly Nagy, József Rácz and István Barcs

摘要

引言：2014 年为匈牙利两次最大的针具交换项目截止年份，该项目虽可能增加注射感染风险，但使可用针具数量缩减一半。2014 年之前匈牙利注射毒品人群（PWIDs）中无男男性行为者（MSM），且该人群未报道新发 HIV 病例。2014、2015 年分别有 1 例和 2 例无男男性行为的注射毒品患者被诊断为 HIV 感染，且感染地可能在匈牙利。2015 年的 2 例患者在艾滋病发病期确诊。此外，2016 年前三个季度在无男男性行为注射毒品人群中报道了 2 例新的（可能是输入性）HIV 病例，其中 1 例被确诊并死于艾滋病。同时，PWIDs 中 HCV 患病率也翻了 1 倍（匈牙利从 24%升至 49%，布达佩斯从 34%升至 61%）。

病例介绍：本文探讨了一例 PWID，于 2015 年 5 月被诊断为 HIV/AIDS，次月死于 AIDS。该例患者未能及时鉴定感染 HIV 病毒，在官方统计中仅作为 PWID HIV 和 PWID AIDS 病例记录，但未统计为 PWID AIDS 死亡病例。虽然容易考虑到疾病后果，但并没有进行随访确

认。据我们所知，医院没有针对意外针刺伤导致艾滋病感染的应急处置预案。
结论：本文提请注意近期报道的 HIV 和 AIDS 监测数据，并认为监测系统运转失灵。虽然仅根据过去两年出现的三例新发 PWID HIV 病例就发出警报还为时尚早，但匈牙利 PWIDs 的艾滋病检测和随访系统肯定存在严重问题。

Translated from English version into Chinese by Peng Song, edited by Pin Yang

Le VIH parmi les consommateurs de drogues injectables en Hongrie

András Ortutay, V. Anna Laetitia, Zsuzsa Marjanek, Károly Nagy, József Rácz et István Barcs

Contexte : Avant 2014 (année de fermeture des deux plus grands programmes d'échange d'aiguilles de Hongrie, qui divisa par deux le nombre de seringues disponibles dans le pays malgré les risques accrus de comportements à risque), aucune infection par le VIH n'avait été rapportée en Hongrie parmi les consommateurs de drogues injectables qui n'étaient pas aussi des hommes ayant des relations sexuelles avec d'autres hommes (HRSH). Un cas en 2014 d'infection par le VIH chez des consommateurs de drogues injectables non HRSH, puis deux en 2015, peut-être contractés en Hongrie, ont été diagnostiqués, les deux cas de 2015 au stade du SIDA. Deux nouveaux cas d'infection de chez des consommateurs de drogues injectables non HSRH (peut-être importés) ont été enregistrés au cours des trois premiers trimestres de 2016, l'un des deux étant par la suite décédé du SIDA. Dans le même temps, la prévalence du VHC a doublé parmi les consommateurs de drogues injectables (de 24 % à 49 % en Hongrie et de 34 % à 61 % à Budapest).
Présentation de cas : Le cas dont traite le présent article est celui d'un homme consommateur de drogues injectables, diagnostiqué séropositif et porteur du SIDA en mai 2015 et décédé du SIDA le mois suivant Son statut d'infection par le VIH a été découvert tardivement et il est apparu dans les statistiques officielles comme un cas incident de VIH chez un toxicomane et un cas incident de SIDA chez un toxicomane, mais pas comme un cas incident de décès du SIDA d'un consommateur de drogues injectables. Il n'y a pas eu de suivi de ses contacts, bien que cela eût été relativement facile compte tenu des circonstances. Il n'existe pas, à notre connaissance, de protocole post-exposition au VIH, en cas d'accident avec exposition au sang, dans les hôpitaux hongrois.
Conclusions : Notre article attire l'attention sur des données récemment publiées de surveillance du VIH et du SIDA et montre l'échec de ce système. Bien qu'il soit peut-être prématuré de donner l'alarme après trois nouveaux cas d'infection par le VIH détectés en deux ans chez des consommateurs de drogues injectables, il existe indéniablement de graves lacunes dans le système de détection et de suivi du VIH parmi les toxicomanes en Hongrie.

Translated from English version into French by Suzanne Assenat, through

ВИЧ-инфицирования среди людей, употребляющих инъекционные наркотики в Венгрии

Андраш Ортутаи, В. Анна Дьярмати, Жужа Марьянек, Карой Надь, Йожеф Рац и Иштван Барч

Предистория: По имеющимся сведениям, до 2014 года (год завершения двух крупнейших программ в Венгрии по обмену игл и шприцов, благодаря которым в стране вдовое сократилось количество доступных шприцов, невзирая на увеличение риска инъекций) в Венгрии не было заявлено о ВИЧ-инфицированных среди наркоманов, употребляющих инъекционные наркотики (НУИН), которые не являлись гомо- или бисексуалами(ММ). В 2014 году был диагностирован один случай, а в 2015 году уже два случая заражения ВИЧ среди НУИН, но не являющимися ММ. Предположительно, заражение произошло в Венгрии. Оба случая заражения в 2015 году были диагностированы на стадии СПИДа. Кроме того, были зарегестрированы еще два новых заражения (хотя это спорный вопрос, т.к. возможно были уже зараженны ранее) в 2016 году так же НУИН, не являющиеся ММ. Одиному был диагностирован СПИД (летальный исход). В то же время распространенность вируса гепатита С (ВГС) удвоилась среди НУИН (с 24% до 49% в Венгрии, и с 34% до 61% в Будапеште).
Презентация дела: Обсуждаемое в данной статье дело касается только НУИН мужского пола, которым был диагностирован ВИЧ и СПИД в мае 2015 года, с летальным исходом от СПИДа в следующем месяце. ВИЧ-инфецирование было обнаружено с опозданием. В официальной статистике случай был обозначен как случай ВИЧ-инфицирования НИУН и случай ВИЧ-инфицирования НИУН СПИДом. СЛучай не был зарегестрирован как ВИЧ-инфицирование НИУН СПИДом с последующим летальный исходом. Никакого отслеживания контактов не последовало, хотя это было бы относительно легко, учитывая обстоятельства. Насколько нам известно, в больницах не существует протокола о заражениях ВИЧ-инфекцией в случае, если заражение ВИЧ произошло из-за возможной травмы от укола иглой.
Выводы: В нашей статье обращается внимание на недавно опубликованные данные о заражения ВИЧ и СПИДом. Так же обращается внимание на несосотятельность системы в этом плане. Возможно, бить тревогу, основываясь на трех новых случаях заражения ВИЧ среди НИУН за последние два года - преждевременное решение. Но, как показывает практика, в системе нужны доработки для выявления и дальнейшего наблюдения за ВИЧ среди НИУН в Венгрии.

Translated from English version into Russian by margarita_, through

TRANSLATORS
WITHOUT BORDERS

El VIH entre las personas que se inyectan drogas en Hungría

András Ortutay, V. Ana Gyarmathy, Zsuzsa Marjanek y Károly Nagy, József Rácz István Barcs

Fondo: Antes de 2014 (año de cierre de los dos programas de intercambio de la aguja más grande en Hungría, que redujo a la mitad el número de jeringuillas disponibles en el país a pesar de

aumentar las prácticas de riesgo de inyección) no se reportó ningún caso de VIH en Hungría entre las personas que se inyectaban drogas (PWIDs), inclusive en hombres que tenían sexo con otros hombres (HSH). En 2014 y 2015 dos no MSM PWIDs fueron diagnosticados recientemente con VIH quienes supuestamente se infectaron en Hungría y ambos casos incidentes de VIH en 2015 fueron diagnosticados en la etapa de SIDA. Además, dos nuevos (aunque supuestamente importados) casos de no MSM PWID se registraron en los tres primeros meses del año 2016, uno de los cuales posteriormente se diagnosticó y luego murió de SIDA. Al mismo tiempo, la prevalencia de VHC (Hepatitis) se duplicó entre PWIDs (de 24% a 49% en Hungría y de 34% a 61% en Budapest).

Presentación del caso: el caso que discutimos en este trabajo es un masculino PWID, que fue diagnosticado con VIH y SIDA en mayo de 2015 y luego murió de SIDA al mes siguiente. El estado de su infección de VIH fue detectado con demora y luego apareció en la estadística oficial como un incidente de un caso PWID VIH y un incidente de un caso de PWID SIDA, pero no como un incidente por muerte PWID SIDA> No se siguió ningún rastreo del contacto, aunque hubiera sido relativamente fácil teniendo en cuenta las circunstancias. En cuanto a lo que nosotros sabemos, no existe ningún protocolo por post exposición a VIH en los hospitales o por una exposición a VIH debido a una lesión del palillo de la aguja final.

CONCLUSIONES: nuestro trabajo llama la atención sobre datos recientemente publicados en cuanto al control de VIH y SIDA y muestra el fracaso del sistema. Mientras que puede ser prematuro que suene la alarma por tres casos recientemente detectados de VIH PWID en los últimos dos años, hay sin duda graves problemas en la detección de VIH y sistema de trazado en PWIDs en Hungría.

Translated from English version into Spanish by Adriana Torres, through

TRANSLATORS
WITHOUT BORDERS
